# Multiscale Progressive Failure Analysis for Composite Stringers Subjected to Compressive Load

**DOI:** 10.3390/ma17133169

**Published:** 2024-06-28

**Authors:** Jian Shi, Jianjiang Zeng, Jie Zheng, Furui Shi, Guang Yang, Mingbo Tong

**Affiliations:** 1 College of Aviation Engineering, Civil Aviation Flight University of China, Guanghan 618307, China; shijian@cafuc.edu.cn; 2 College of Aerospace Engineering, Nanjing University of Aeronautics and Astronautics, Nanjing 210016, China; 3 Department of Mechanical Engineering, University of Alberta, Edmonton, AB T6G 2R3, Canada; furui@ualberta.ca; 4 AML, Department of Engineering Mechanics, Tsinghua University, Beijing 100084, China

**Keywords:** composite stringer, matrix damage, multiscale analysis, GMC

## Abstract

The fiber-reinforced composite stringer is commonly used in large civil aircraft wing structures. Under compression loads, it exhibits complex failure modes, with matrix cracking being one of the most common. The quantitative analysis of matrix failure is important and difficult. To address this issue, a multiscale method combining the generalized method of cells (GMC) and macroscopic FEM models is employed to quantitatively predict matrix damage and failure. The extent of matrix damage in the composite structure is represented by the number of failed matrix subcells within the repeating unit cells. The 3D Tsai–Hill failure criterion is established for the matrix phase, and the maximum stress failure criterion is applied to the fiber subcell. Upon meeting the criterion, the stiffnesses of the failed subcells are immediately reduced to a nominal value. In the current study, the ultimate loads, failure modes and load–displacement curves of composite stringers subjected to compressive load are obtained by the experiment approach and the proposed multiscale model. The experimental and simulation results show good agreement, and the multiscale analysis method successfully predicts the extent of matrix damage in the composite stringer under compressive load. The number of failed matrix subcells quantitatively evaluates the damage extent within a 2 × 2 GMC model. The findings reveal that matrix subcell failures primarily occur in the 45° and −45° plies of the middle part of the stringer composite.

## 1. Introduction

Composite stringers are widely used in the wing structures of large civil aircraft [1,2]. Under compression loads during flight, they exhibit complex failure modes, making this a focal point in many studies [3,4,5,6,7,8,9]. For example, Vescovini and Bisagni [10] studied the local buckling problem for stiffened composite stringers with different cross sections, such as cap, J, T and I-shaped, under compression and shear loads using a genetic optimization algorithm and semi-analytical method. Wang et al. [11] conducted global and local buckling analysis of the stiffened composite plate. In another study, Song et al. [12] studied the damage and failure mechanism of the interface between the stringer and the skin in the composite structure under hail impact at different speeds by employing a rate-sensitive ice model. Cosentino et al. [13] proposed a nonlinear analysis method for debonding in skin/stringer composite assemblies. This method employed a Rayleigh–Ritz approach based on Galerkin’s orthogonal eigenfunctions, and the results show a fairly good correlation with reported experimental data. Yang et al. [14] investigated the buckling loads of hat-stiffened composite panels subjected to axial compression loading by exploiting several engineering calculation methods. The axial stiffness of the stringer and skin matched favorably with experimental data. Chen [15] predicted the ultimate strength and progressive failure of sandwich composite hat-stringer-stiffened panels under transverse in-plane loads, achieving more accurate results. Li et al. [16] studied the static and fatigue failure of hat-stringer-stiffened composite panels under four-point bending using a novel theoretical model. Most research [10,11,12,13,14,15,16] on the failure, buckling, bending resistance, and other properties of composite stringers relies on macroscopic analysis methods. There are relatively few quantitative analyses of the failure of composite stringers using multiscale analysis [17,18]. In the current study, multiscale numerical simulations and experimental analyses are conducted on composite stringers of civil aircraft under compressive loads.

The multiscale analysis method can combine the high efficiency of macroscopic analysis with the high precision of microscopic analysis, which has become an important research field in the analysis of composite structures [17,18,19]. Paley and Aboudi [20] first proposed the general method of cells (GMC) micromechanics model. This model was later refined by taking stresses as the basic unknown variables in order to improve computational efficiency [21]. GMC is an analytical method in nature and can be easily embedded into the integral points of FEM models to form a high-efficiency and high-precision multiscale calculation approach. The GMC model is widely applied to analyze fiber-reinforced and woven composites [22,23,24,25]. For example, Paria et al. [26] performed failure analysis of notched and unnotched laminates under both tensile and compressive static loading using the generalized method of cells; it has been verified that the GMC analysis method is highly efficient and accurate. May and Hiermaier [27] proposed a computationally efficient concurrent multiscale methodology that combined the GMC and the rule of mixtures. This methodology was verified using one-element tests on unidirectional composites and validated against high-velocity impact experiments. Shi et al. [28,29] carried out multiscale progressive damage analysis on the open-hole plates taken from composite stringers based on the GMC method, and the analysis results agree well with the test results. Pineda et al. [30] proposed a multiscale physics method based on GMC for laminated composite structures and quantitatively analyzed the matrix failure of the 2D FEM model.

This paper presents multiscale numerical and experimental studies on composite stringers of civil aircraft under compressive loads. The multiscale method based on GMC is implemented through a user-defined subroutine (UMAT) in Abaqus 2022 software to predict the load–displacement curve, ultimate load, and failure modes of fiber-reinforced composite structures. The analysis results are then compared with the test results. Given the excellent predictive ability of the GMC, a quantitative analysis of matrix damage and failure for the 3D stringer FEM subjected to compressive loads is further investigated.

## 2. Multiscale Progressive Damage Model

In the current study, microscopic failure criteria are established based on the GMC model, which is applied to the constituent materials of composite laminate structures. The fiber constituent is generally believed to be linearly elastic and brittle before fiber failure occurs, so the maximum stress criterion [31] can be adopted for the fiber constituent.

(1) $${\left(\frac{\sigma_{11}^{\left(\beta_{f}\gamma_{f}\right)}}{X_{ft}}\right)}^{2}=d_{f}^{2},\;\sigma_{11}^{\left(\beta_{f}\gamma_{f}\right)}>0$$

(2) $${\left(\frac{\sigma_{11}^{\left(\beta_{f}\gamma_{f}\right)}}{X_{fc}}\right)}^{2}=d_{f}^{2},\;\sigma_{11}^{\left(\beta_{f}\gamma_{f}\right)}<0$$

where *β_f_ γ_f_* represents the index number of the fiber subcell for the GMC; *X_ft_* and *X_fc_* are the tensile and compressive strength of the fiber constituent, respectively; and $\sigma_{11}^{\left(\beta_{f}\gamma_{f}\right)}$ denotes the axial stress component of the fiber. Once the damage variable *d_f_* in Equations (1) and (2) is equal to or greater than 1, the corresponding fiber subcell meets the failure criterion, after which the stiffness matrices of the failed fiber subcells are reduced to 0.01% of the initial value.

The 3D Tsai–Hill failure criterion [32] is used to predict the failure of the matrix phase, the matrix constituent is assumed to be isotropic, and its formula is as follows:

(3) $$\begin{array}{l} \frac{{\left(\sigma_{11}^{\left(\beta_{m}\gamma_{m}\right)}\right)}^{2}+{\left(\sigma_{22}^{\left(\beta_{m}\gamma_{m}\right)}\right)}^{2}+{\left(\sigma_{33}^{\left(\beta_{m}\gamma_{m}\right)}\right)}^{2}}{Y_{mt}^{2}}+\frac{-\sigma_{11}^{\left(\beta_{m}\gamma_{m}\right)}\sigma_{22}^{\left(\beta_{m}\gamma_{m}\right)}-\sigma_{11}^{\left(\beta_{m}\gamma_{m}\right)}{\left(\sigma_{33}^{\left(\beta_{m}\gamma_{m}\right)}\right)}^{2}-\sigma_{22}^{\left(\beta_{m}\gamma_{m}\right)}\sigma_{33}^{\left(\beta_{m}\gamma_{m}\right)}}{Y_{mt}^{2}}\\ +\frac{{\left(\sigma_{12}^{\left(\beta_{m}\gamma_{m}\right)}\right)}^{2}+{\left(\sigma_{13}^{\left(\beta_{m}\gamma_{m}\right)}\right)}^{2}+{\left(\sigma_{23}^{\left(\beta_{m}\gamma_{m}\right)}\right)}^{2}}{T_{m}^{2}}=d_{m}^{2},\;{\bar{\sigma }}_{22}>0 \end{array}$$

(4) $$\begin{array}{l} \frac{{\left(\sigma_{11}^{\left(\beta_{m}\gamma_{m}\right)}\right)}^{2}+{\left(\sigma_{22}^{\left(\beta_{m}\gamma_{m}\right)}\right)}^{2}+{\left(\sigma_{33}^{\left(\beta_{m}\gamma_{m}\right)}\right)}^{2}}{Y_{mc}^{2}}+\frac{-\sigma_{11}^{\left(\beta_{m}\gamma_{m}\right)}\sigma_{22}^{\left(\beta_{m}\gamma_{m}\right)}-\sigma_{11}^{\left(\beta_{m}\gamma_{m}\right)}{\left(\sigma_{33}^{\left(\beta_{m}\gamma_{m}\right)}\right)}^{2}-\sigma_{22}^{\left(\beta_{m}\gamma_{m}\right)}\sigma_{33}^{\left(\beta_{m}\gamma_{m}\right)}}{Y_{mc}^{2}}\\ +\frac{{\left(\sigma_{12}^{\left(\beta_{m}\gamma_{m}\right)}\right)}^{2}+{\left(\sigma_{13}^{\left(\beta_{m}\gamma_{m}\right)}\right)}^{2}+{\left(\sigma_{23}^{\left(\beta_{m}\gamma_{m}\right)}\right)}^{2}}{T_{m}^{2}}=d_{m}^{2},\;{\bar{\sigma }}_{22}<0 \end{array}$$

where *Y_mt_*, *Y_mc_* and *T_m_* are the transverse tensile strength, transverse compressive strength and shear strength of the matrix subcell, respectively. When the damage variable *d_m_* in Equations (3) and (4) is equal to or greater than 1, the corresponding matrix subcells satisfy the failure criterion and have failed. Then, the stiffness of the failed matrix subcells are reduced to 0.01% of the initial value.

**Figure 1:**
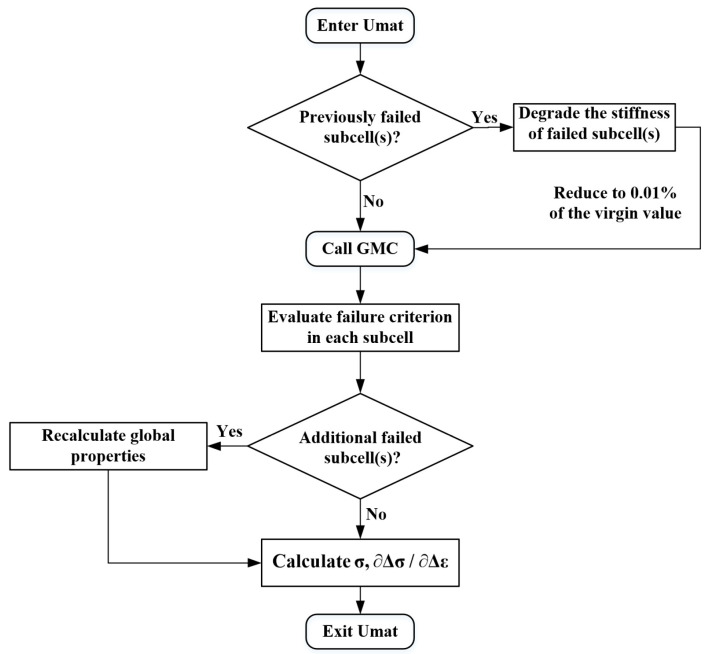
Multiscale algorithm flow chart based on GMC.

At every integral point of each element, the calculation of the micromechanics module at each incremental step is realized by calling the UMAT subroutine, which integrates the microscopic failure criterion and GMC algorithm. Figure 1 shows the flow diagram of the multiscale algorithm based on the GMC model, including the microscopic failure criterion and its corresponding damage evolution laws. 

The strain field at the finite element integration points, calculated and updated in Abaqus/Standard, serves as the basic macro load condition for the GMC model [21,30]. The GMC model calculates the stress and strain fields for each subcell. The state variable records the failed subcell of the previous incremental step and transfers it to the current incremental step. Next, the re-judgment process of the failed subcell by the micro failure criterion is skipped. At the same time, the presence of new failure subcells at the current incremental analysis step is determined based on the microscopic failure criteria. If new failures are identified, a new state variable is recorded and passed to the next incremental step. Once a subcell fails, its stiffness matrix is immediately reduced, and its contribution to the GMC model is almost negligible. The stiffness matrix of the entire GMC model is updated and returned to the integration point for the next iteration calculation until the multiscale calculation is completed.

## 3. Uniaxial Compression Experiments of Stringer Composite

The stringer composite structure comprises four parts: upper flange, web, lower flange and skin. The lower flange and skin are bonded and can be regarded as a whole thickened laminated plate. The stringer specimen is 200 mm long, with 50 mm at each end serving as the strengthening parts and 100 mm in the middle designated as the test and examination section. The geometric sizes of the stringer mainly include the width and thickness of each section of the four parts, where the thickness of each plate is obtained by multiplying the thickness of a single layer by the number of layers. The thickness of each single layer is 0.19 mm. The section width, fiber angle and layer number are shown in Table 1.

**Figure 2:**
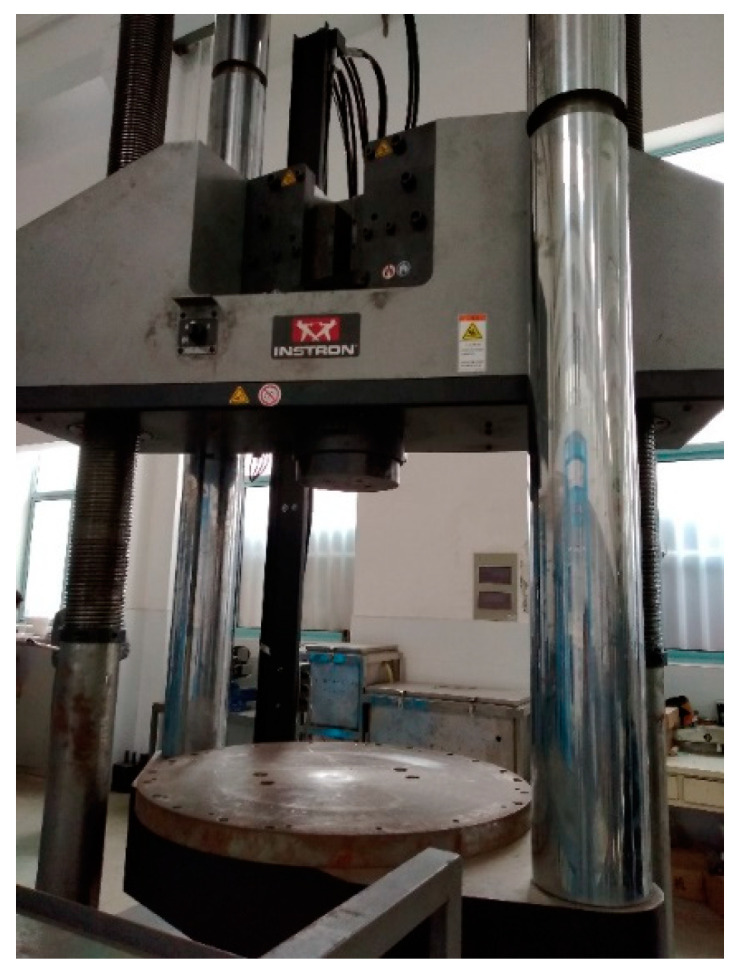
The INSTRON test machine.

The uniaxial compression experiments of the H-shaped stringer specimens were carried out on the INSTRON test machine shown in Figure 2. The test machine has a maximum carrying load of 1500 kN and a load accuracy of 1%. Its height and width are about 3.5 m and 0.8 m, respectively. One end of the specimen is fixed, and the other is subjected to a downward displacement load during the tests.

**Figure 3:**
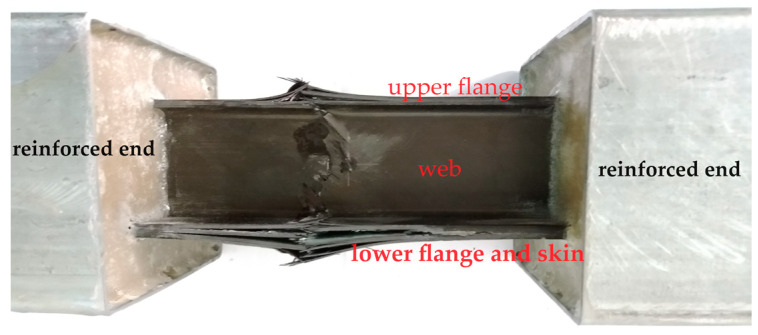
The final failure state of the stringer.

In the experiment, the failure of the stringer begins from the middle of the upper flange and extends along the web to the lower upper flange and the skin. The failure starting from the middle of the specimen indicates that, when using the fixed sealing reinforcement parts, the constraint conditions and the loading process are reasonable. This approach avoids stress concentration near the fixed sealing end due to unbalanced loading. The failure state of the stringer is shown in Figure 3.

The failure starts from the middle of the upper flange with the smallest section size and the least number of layers, and the damage is gradually transmitted to the web and lower flange. The failure in the middle of the upper flange causes the test specimen to bend upward to a certain extent. This is because the bearing capacity of the upper flange is relatively weak, and the damage degree is the largest, which results in local crushing. Ultimately, the lower flange and skin are damaged or fail. During the compression process, the 0° layer plays the main bearing role, and the process of fiber fracture and matrix failure is accompanied by delamination for many composite layers.

## 4. Finite Element Model Description

**Figure 4:**
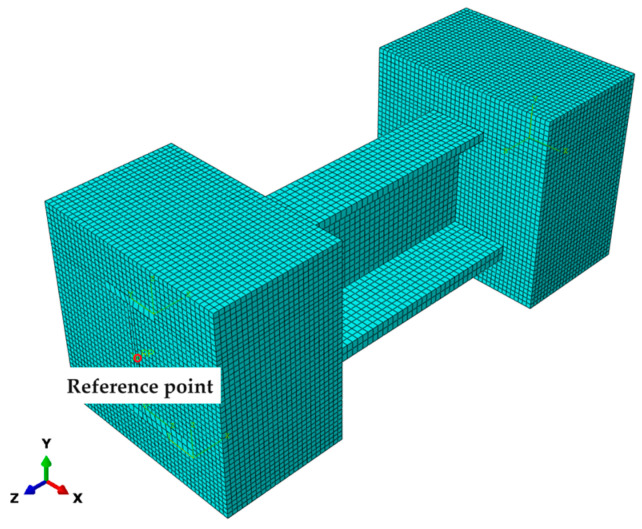
FEM mesh of stringer structure.

The element type used in the 3D finite element model of the stringer is C3D8R. One end of the specimen is fixed, while the other is subjected to a displacement load. A reference point is set at the geometric center of the stringer’s loading surface to ensure that the loads acting on the end face nodes are more uniform and reasonable. A multi-point constraint (MPC) is applied, which ties the reference point to all nodes of the loading surface. The displacement load of 3 mm along the axial direction of the stringer is applied to the reference points, making it convenient to monitor and output the load–displacement historical data during the multiscale progressive damage analysis. The middle part of the stringer is the focus of the analysis, and the FEM of the stringer structure is shown in Figure 4.

The layup attributes of the stringer finite element model are assigned according to Table 1 above. The composite material used is T800S/3900-2B, which is widely equipped with the main bearing structure of advanced civil aircraft. Table 2 and Table 3 show the elastic modulus and strength parameters of the single layer provided by vendors. The material parameters of the fiber and matrix at the microscopic level are adjusted using the GMC model, as shown in Table 4 and Table 5.

**Figure 5:**
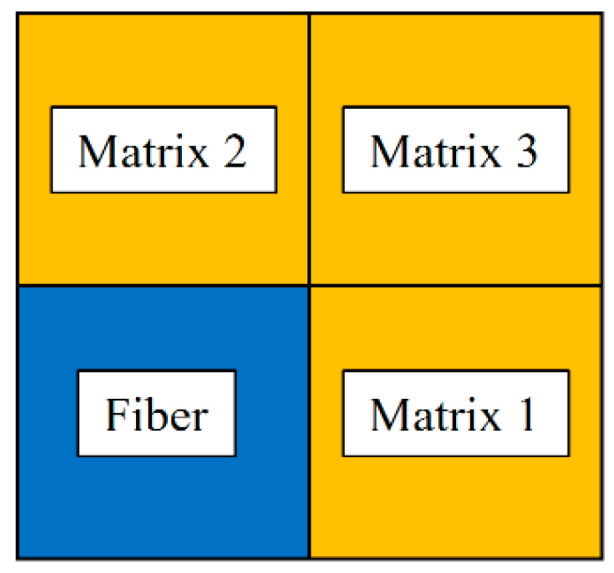
2 × 2 GMC used in multiscale simulations.

A square-shaped repeating unit cell with a size of 2 × 2 without considering the interfacial phase was selected, including three matrix cells (colored orange) and one fiber cell (colored blue), as shown in Figure 5. The square-shaped GMC model of 2 × 2 scale is embedded on the integral point of each stringer element, and the above proposed multiscale progressive damage analysis model is adopted for its subcells. The progressive damage process of this GMC model consists of three parts: damage initiation, progressive damage evolution and final failure. The matrix or fiber subcells are considered linearly elastic before damage occurs. The stiffness matrix of the subcell is directly reduced after the micro failure criterion is met.

The GMC model embeds the same integration point that resolves the macro strain to the subcells. The stresses and strains of each matrix subcell vary, resulting in different damage evolution degrees or failure states for the three matrix subcells. Consequently, the multiscale method can more accurately describe the damage and failure states than the macroscopic method. For example, to determine the failure of a composite structure in the FEM model at the macro level, an element may exhibit a certain form of matrix failure, indicating that all matrix constituents have failed. However, the GMC model can quantify the degree of matrix constituent failure more precisely, showing whether only a portion of the matrix subcells or all subcells have failed. The stress fields, strain fields, or failure states of matrix subcells at the micro scale can be output for the concerned region in the finite element model. This allows one to visualize the matrix phase damage degree at each structure element, enabling better quantitative judgment and evaluation.

## 5. Results and Discussion

**Figure 6:**
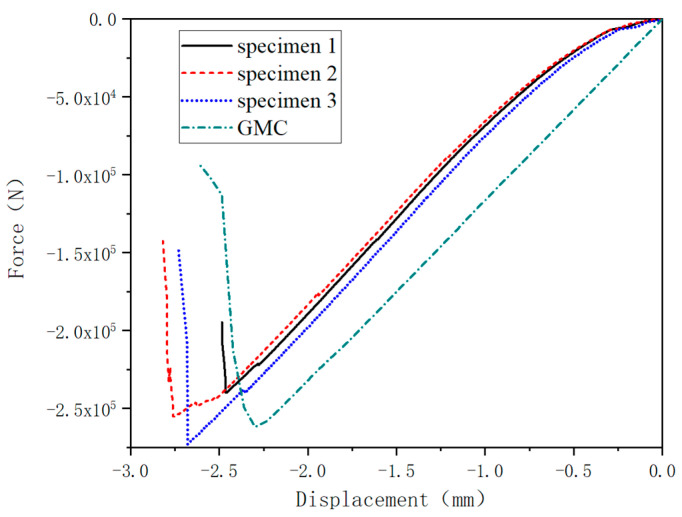
Load versus displacement of stringer.

The experiments tested three stringer specimens. Figure 6 shows the displacement and load curves obtained from the tests and the multiscale simulation analysis. In the initial stage of the test, the structural stiffness of the test specimen is lower, because the two reinforced ends of the stringer primarily bear the load. With the increase of the displacement load, the reinforced part may produce some small cracks, and the position for bearing force is gradually transferred to the composite structure, making the stiffness of the displacement versus load curve gradually increase and return to normal. The average failure load of the test specimen is 257.2 kN.

The load versus displacement curve obtained from the simulation does not exhibit obvious nonlinear behavior. This is because the matrix contributes little to the stiffness of the composite stringer. In contrast, the fibers in the 0° layer contribute the most and exhibit linear–elastic and brittle characteristics. Additionally, the FEM model of the stringer structure does not consider the cohesive effect between composite layers to simulate delamination failure. The simulated ultimate load is 259.7 kN, and the error coefficient compared with the experimental data is around 1%.

The subcell failures of the 2 × 2 sized GMC model are represented by the solution dependent state variables (SDV) within the Abaqus 2022 UMAT subroutine. SDV1 to SDV4 indicate whether subcells 1 to 4 have failed. An SDV value of 0 indicates that the corresponding subcell has not failed, while an SDV value of 1 indicates that subcell failure has occurred. SDV5 is set to an integer from 0 to 3, which represents the number of matrix subcell failures in the GMC model. This allows for the quantitative analysis of matrix failure in the finite element model.

**Figure 7:**
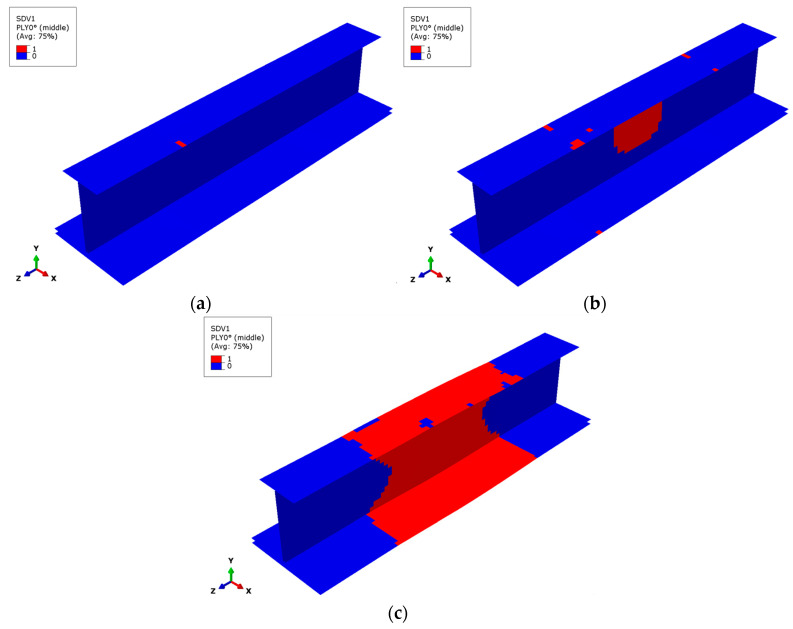
Fiber subcell failure in the outermost 0° layer. (**a**) Fiber initial failure, (**b**) fiber failure at the ultimate load, (**c**) fiber final failure.

As the GMC model used in this study includes only one square fiber subcell, the failure and expansion states of this fiber subcell represent the respective fiber failure and expansion in the macro-level finite element model. Figure 7 shows the failure expansion of the fiber subcell in the outermost 0° layer of the composite stringer. The initial failure of the fiber subcell occurs in the area where the upper and lower flanges contact the reinforced end (see Figure 7a). The contact constraint conditions set in this area easily cause stress concentration, leading to the failure of the fiber subcell. At this point, the external load is very close to the ultimate load of the specimen. Under the ultimate load, a large area of fiber subcell failure occurs in the middle part of the web (see Figure 7b). With the continuous application of displacement load, the failure area of the fiber subcells expands rapidly. Simultaneously, the connection point between the middle of the upper flange and the web fails (see Figure 7c). This occurs because the web is prone to geometric buckling deformation and stress concentration. Its ply number and thickness are relatively small, resulting in a weaker bearing capacity. After local fiber fracture occurs, the maximum stress location and force transmission path quickly transfer to the top and lower flange. The failure results obtained by the simulation are consistent with the test results.

**Figure 8:**
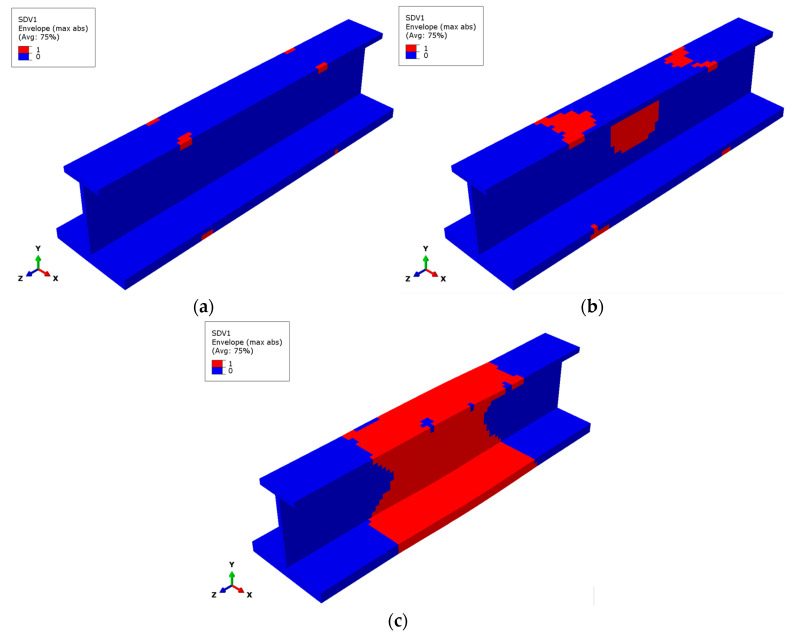
Fiber subcell failure for the stringer composite. (**a**) Fiber initial failure, (**b**) fiber failure at the ultimate load, (**c**) fiber final failure.

Figure 8 shows the situation of fiber subcell failure in the composite stringer. It can be seen that the initial failure of the fiber subcell occurs at the place where the upper flange is in contact with the reinforced end (see Figure 8a). At the ultimate load, fiber failure concentrates in the middle of the web and on the upper flange near the reinforced end (see Figure 8b). In contrast, the final failure area of fiber subcells concentrates in the middle of the specimen. The overall states of fiber subcell failure are very close to the failure states of the outermost 0° layer (see Figure 8c), indicating that fiber subcell failure of the stringer composite mainly occurs in the 0° layer, and that the fiber failure states in different 0° layers are relatively consistent.

**Figure 9:**
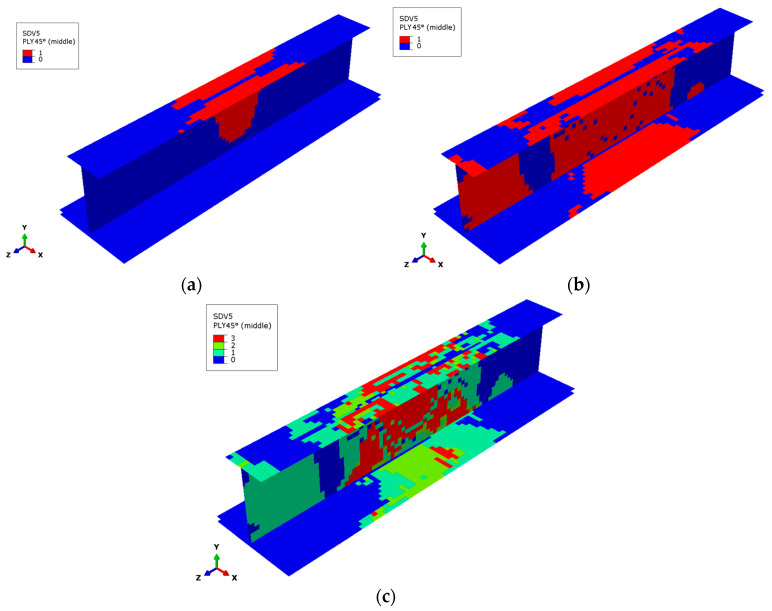
Matrix subcell failure in the outermost 45° layer. (**a**) Matrix subcell initial failure, (**b**) matrix subcell failure at the ultimate load, (**c**) matrix subcell final failure.

**Figure 10:**
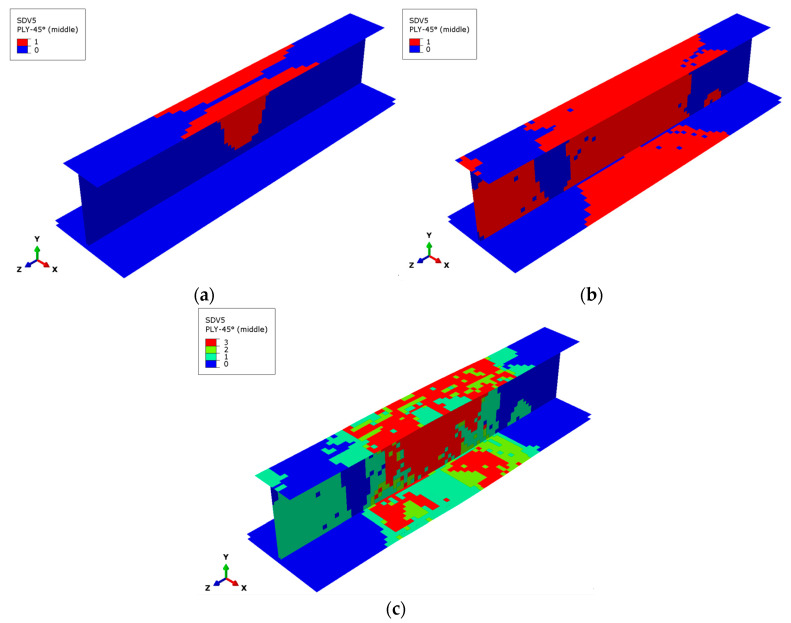
Matrix subcell failure in the outermost −45° layer. (**a**) Matrix subcell initial failure, (**b**) matrix subcell failure at the ultimate load, (**c**) matrix subcell final failure.

Figure 9 and Figure 10 show the failure states of the matrix subcells of the 45° and −45° plies at the outermost side of the stringer, respectively. The failure states of the three matrix subcells in the GMC model are different, making it easy to judge the degree of matrix damage and failure for the finite element model. The failure of the matrix subcells mainly occurs in the 45° and −45° plies, and the failure distributions of the two plies are similar. The following introduces the matrix subcell failure of the 45° ply.

Figure 9a shows the failure state of the matrix subcells of the 45° layup under 200 kN load. The matrix failure occurs at the upper flange in the middle of the web, and just one matrix subcell fails at this load. Figure 9b shows the failure of the matrix subcells under the ultimate load. A large area of single matrix subcell failure occurs in the upper part of the upper flange and the middle part of the web. Figure 9c shows the final failure state of matrix subcells for the 45° layup. In the finite element model, the red area represents the failure of all matrix subcells, the yellow elements represent the failure of two matrix subcells, and the green part represents the failure of only one matrix subcell. The blue part indicates that no matrix subcell failure appears. The final failure state of the matrix subcells in the 45° layer is distributed irregularly; there are some areas where all three matrix subcells failed in the upper flange and the middle of the web. The matrix subcell failure of the −45° layer shown in Figure 10 is relatively similar to that of the 45° layer.

**Figure 11:**
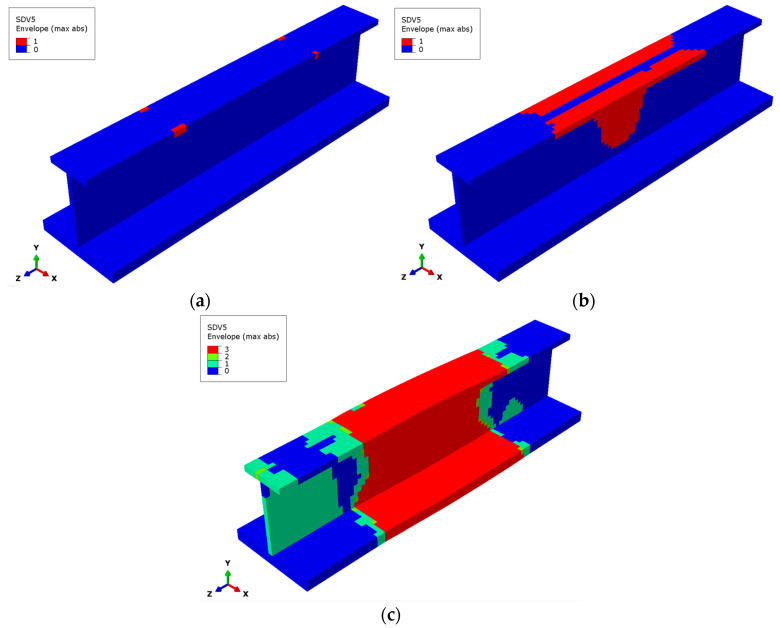
Matrix subcell failure for the stringer composite. (**a**) Matrix subcell initial failure, (**b**) matrix subcell failure at the ultimate load, (**c**) matrix subcell final failure.

Figure 11 shows the matrix subcell failure envelope in (a) the initial phase under a 200 kN load, (b) the ultimate load, and (c) the final load for the stringer. It can be observed that the overall failure states are the failure superposition of the 45° and −45° layers, proving that the failure of the matrix subcells mainly occurs in these two layers. The areas where the two-matrix subcell failure occurs simultaneously are concentrated in the middle of the upper and lower flange, mainly as a transition between the single-matrix subcell failure area and the three-matrix subcell failure area. The area of single-matrix subcell failure occupies a relatively large proportion, and the position is close to both ends. There is a relatively large intact area without matrix damage at both ends of the stringer.

**Figure 12:**
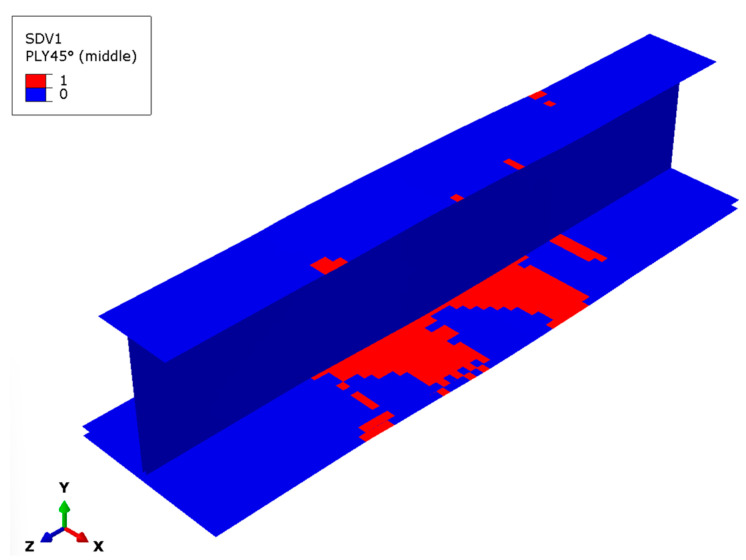
Fiber subcell failure in the outermost 45° layer.

For the fiber subcells in the 45° or −45° layers, some fiber subcells do not fail until the final load is applied, specifically in the middle part of the lower flange. The fiber subcell failures in the 45° layer, as shown in Figure 12, are limited to some irregular and small-scale compression failure areas scattered on the lower flange. Most fiber subcells in these elements remain intact, contrasting sharply with the failure states of the fiber subcells in the 0° layer.

**Figure 13:**
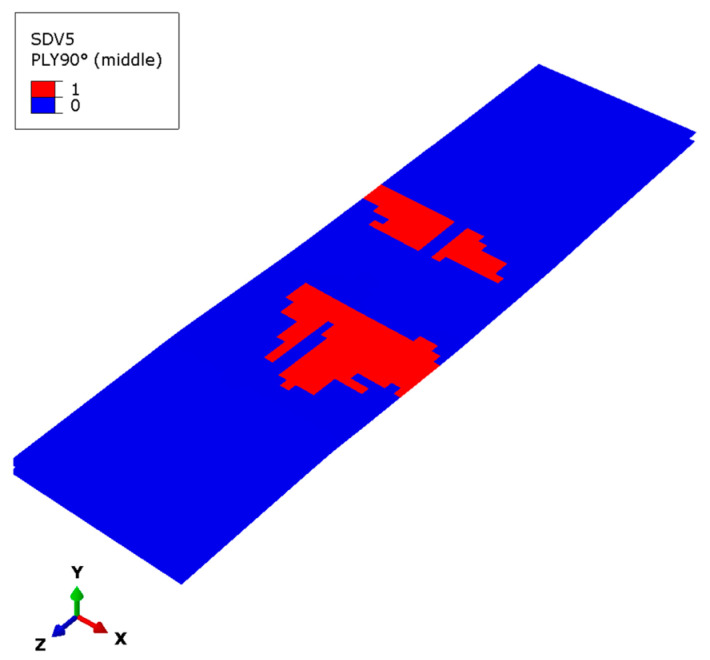
Matrix subcell failure in the outermost 90° layer of the lower flange.

Figure 13 shows the failure state of the matrix subcells in the outermost 90° ply, which is only present at the lower flange. The failed position is mainly located in the middle of the lower flange. After the upper flange and web fail, the main external load is transferred to the lower flange. The 0° layer and ±45° layer bear the greater force, and their damage occurs first. Consequently, the matrix subcell failure of the 90° layer occurs last and its failure area is relatively small.

**Figure 14:**
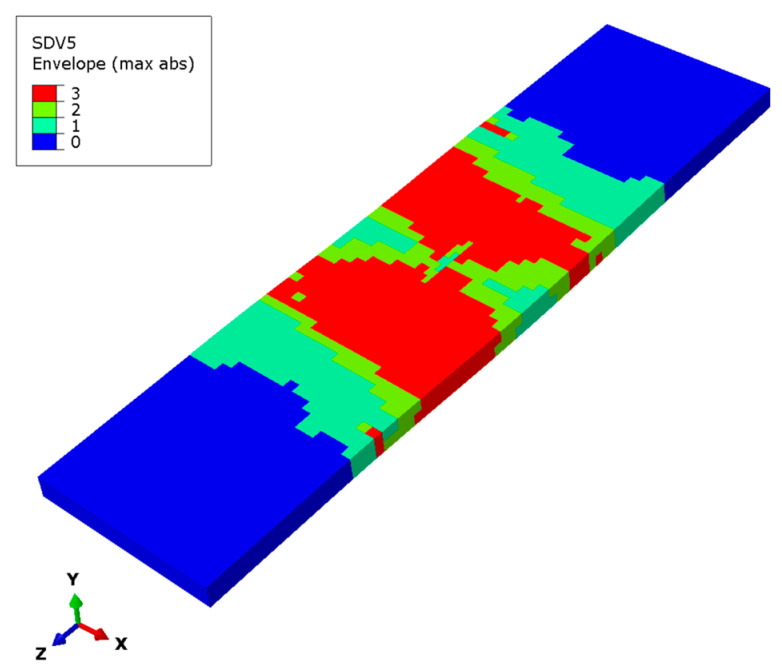
Matrix subcell failure for the lower flange.

Figure 14 presents the overall matrix subcell failure envelope for the lower flange laminates. Comparing the two failure states above for the lower flange reveals that the matrix subcell failures of these laminates mainly occur in the 45° and −45° plies. The red area indicates that all three matrix subcells failed simultaneously, superimposing the failure of the matrix subcells of all plies, and is mainly concentrated in the middle of the lower edge. There is no damage at either end of the lower flange laminates, which proves that its bearing capacity is stronger for the composite stringer.

## 6. Conclusions

The ultimate loads, failure states, and load versus displacement curves of H-shaped stringer composites were investigated through compressive tests and multiscale simulation analysis. Three specimens were tested, with an average ultimate load of 257.2 kN. The failure modes were mainly fiber and matrix compressive failures concentrated in the middle of the specimens. The multiscale progressive damage and failure analysis, based on a 2 × 2-sized GMC micromechanical model that employs the 3D Tsai–Hill criterion for the matrix phase and the maximum failure criterion for the fiber constituent, is used to analyze the damage and failure process of the composite stringer. The following conclusions are obtained:

(1) The results show that fiber subcell failure in the composite stringer mainly occurs in the 0° layer, with relatively consistent fiber failure states across different 0° layers. The failure of the matrix subcells predominantly occurs in the 45° and −45° plies, with the failure distributions of these two plies being essentially similar.

(2) The results of ultimate load and failure modes obtained by experiment and simulation show good agreement. The multiscale analysis model accurately predicts the failure extent of the matrix subcells for different layers, which realizes the quantified analysis for the matrix failure of the stringer composite structure.

## Figures and Tables

**Table 1:** The section size and ply angles of the stringer.

Section Size (mm)	Ply Angles	Numbers of Plies
Upper flange = 24	[45/−45/0/0/−45/0/45/0/0/0/45/0/−45/0/0/−45/45]	17
Web = 40	[45/−45/0/0/−45/0/45/45/0/−45/0/0/−45/45]	14
Lower flange = 50	[45/−45/0/0/45/0/−45/0/0/−45/45]	11
Skin = 50	[45/90/−45/0/90/−45/45/0/−45/0/0/−45/0/45/ −45/90/0/−45/90/45]	20

**Table 2:** Mechanical properties of the unidirectional lamina.

*E*_1_/GPa	*E*_2_/Gpa	*E*_3_/Gpa	*G*_12_/Gpa	*G*_13_/Gpa	*G*_23_/Gpa	*ν*_12_	*ν*_13_	*ν*_23_
154	9	9	7.0	7.0	7.0	0.29	0.29	0.45

**Table 3:** Strength properties of the unidirectional lamina.

*X_T_*/Mpa	*X_C_*/Mpa	*Y_T_*/MPa	*Y_C_*/Mpa	*Z_T_*/Mpa	*Z_C_*/Mpa	*S*_12_/Mpa	*S*_13_/MPa	*S*_23_/MPa
2690	1380	93	211	93	211	109	109	109

**Table 4:** Elastic properties of the composite constituents.

Fiber Properties	Value (Gpa)	Matrix Properties	Value (Mpa)
Longitudinal modulus *E_f_*_11_	292	Modulus *E_m_*	2000
Transverse modulus *E_f_*_22_	91	Poisson’s ratio *v_m_*	0.35
Major Poisson’s ratio *v_f_*_12_	0.23	Shear modulus *G_m_*	2300
Transverse shear modulus *v_f_*_23_	0.45	Fiber volume fraction *v_f_*	0.52
In-plane shear modulus *G_f_*_12_	55		

**Table 5:** Strengths of the composite constituents.

Strength	Value (Mpa)
*X_ft_*	Tensile strength of the fiber 7586
*X_fc_*	Compressive strength of the fiber 2855
*Y_mt_*	Tensile strength of the matrix 49
*Y_mc_*	Compressive strength of the matrix 124
*Y_m_*	Matrix shear modulus 41

## Data Availability

The raw data supporting the conclusions of this article will be made available by the authors on request.
